# The impact of occupational hazards and traumatic events among Belgian emergency physicians

**DOI:** 10.1186/s13049-016-0249-9

**Published:** 2016-04-27

**Authors:** Francis J. Somville, Véronique De Gucht, Stan Maes

**Affiliations:** Department of Health and Medical Psychology, University of Leiden, Wassenaarseweg 52, Leiden, The Netherlands; Department of Emergency and Traumatology, AZ St Dimpna, J.B. Stessenstraat 2, Geel, 2440 Belgium

**Keywords:** Emergency physicians, Job satisfaction, Occupational hazards, Post-traumatic stress, Professional burnout, Social support

## Abstract

**Background:**

Emergency Physicians (EPs) are regularly confronted with work related traumatic events and hectic work conditions. Several studies mention a high incidence of post-traumatic stress disorder (PTSD) and psychosomatic complaints in EP.

The main objective of this study is to examine the contribution of demographics, traumatic events, life events, the occurrence of occupational hazards and social support to post-traumatic stress symptoms (PTSS), psychological distress, fatigue, somatic complaints and job satisfaction in Emergency Physicians.

**Methods:**

For this study questionnaires were distributed to Belgian Emergency Physicians, These include, as determinants socio-demographic characteristics, traumatic events, life events, the occurrence of physical hazards, occurrences of violence, occurrence of situations that increase the risk of burnout and social support by supervisors and colleagues (LQWQ-Med), and as outcomes PTSS (IES), psychological distress (BSI), somatic complaints (PHQ 15), perceived fatigue (CIS20 R) and job satisfaction (LQWQ-MD). The response rate was 52.3 %. Hierarchical multiple regression analysis was performed to examine the association between the determinants and each of the outcomes.

**Results:**

Emergency Physicians are particularly vulnerable to post-traumatic and chronic stress consequences due to repetitive exposure to work related traumatic incidents such as serious injuries or death of a child/adolescent. One out of three Emergency Physicians met sub-clinical levels of anxiety and 14.5 % met a clinical level of PTSD, short for Post-Traumatic Stress Disorder. Levels of fatigue were high but not directly related to traumatic events and occupational hazards. Social support from colleagues was found to have a beneficial effect on these complaints. Job satisfaction seems to have a protective factor. All of these not only affect the Emergency Physicians themselves, but can also have an adverse impact on patient care.

**Discussion:**

EPs are, according to our and other studies, confronted on a regular basis with significant, potentially traumatizing work related events. There is a higher perception of traumatic events in older Eps. We find out that 36 % of the EPs find dealing with sudden death of a young person and traumatic accident/disease involving a young person the most traumatic experience during their work activity. Three quarter of these EPs have children of their own. The results of the study show that frequency of exposure to traumatic (work) events contributes next to occurrence of situations that increase the risk of burnout to the explanation of variance in posttraumatic stress and psychological distress. The novelty of this study is that it explores the effect of specific determinants of PTSS, psychological distress, fatigue, somatic complaints and job satisfaction in Emergency Physicians. Especially occurrence of situations that increase the risk of burnout seems to have a major impact on all outcomes including job satisfaction, while occurrence of violence contributes especially to psychological distress and perceived fatigue. Lack of social support is a well-known predictor of occupational stress in emergency care workers. In contrast however, good social support of colleagues at work, as we found in our study, can facilitate the recovery process after confrontation with traumatic events and occupational hazards.

**Conclusion:**

Emergency Physicians are particularly vulnerable to post-traumatic stress and chronic stress consequences due to repetitive exposure to work related traumatic events. Training in dealing with violence and situations that can increase the risk of burnout can reduce detrimental consequences in emergency physicians. In addition, it is suggested that emergency units are screened systematically on determinants of burnout, in view of interventions. Finally, creating a supportive work environment and training the medical staff in supportive skills with backup by experts may also reduce adverse consequences of confrontation with traumatic work events.

## Background

Emergency physicians are significantly at risk due to increased exposure to work-related traumatic events than other physicians [1, 2]. Emergency professionals have a risk of exposure to these events due to the nature of emergency medicine. The most traumatic events are: sudden infant death syndrome (SIDS); traumatic incidents involving children; dealing with patients’ relatives and family; handling burn victims and confrontation with psychiatric patients [3, 4].

In addition, repetitive overexposure to other occupational hazards such as physical hazards, violence, and situations that increase the risk of burnout may also have important health and wellbeing consequences in Emergency Physicians [5]. These occupational hazards include blood borne pathogens; non-blood borne pathogens as latex allergy, radiation exposure, nitrous oxide inhalation, rotating shift work, violence and burnout [6]. Confrontation with violence is a common problem in an emergency medicine department. In one study more than half of the staff members reported that they were exposed to verbal violence in the past year and 8.5 % to physical violence [7]. In another study, more than 60 % reported high levels of burnout symptoms, which is much higher than in other physicians [8]. Post-traumatic stress symptoms (PTSS) and Post-traumatic stress Disorder (PTSD) are common among rescue and ambulance personnel [9–13]. The incidence of PTSD is found to be much higher in emergency physicians (EPs) than in other physicians and even emergency nurses [14–16]. One of the reasons for this may be a difference in exposure to traumatic events and occupational hazards. It is well known that exposure to traumatic events and/or occupational hazards may have a whole range of psychological consequences such as nightmares, recurrent thoughts, flashbacks, sleeping problems, irritability, depression, lack of interest in daily life, anger, loss of concentration, restlessness, burnout and clinical levels of depression. Lack of adequate social support may contribute to the aggravation and persistence of these consequences [10, 15, 17, 18]. These stress consequences may also cause reduced job satisfaction and commitment, absenteeism and turnover in emergency care personnel and negatively influence the quality of care [6, 12].

The main aim of the present study is to examine the contribution of demographic characteristics, frequency of confrontation with work related traumatic events, occurrence of life events, the occurrence of occupational physical hazards, psychosocial occupational hazards (violence and situations that increase the risk of burnout) and perceived social support to: PTSD, psychological distress (anxiety and depression), somatic complaints, fatigue and job satisfaction in Emergency Physicians. With the exception of age and social support, all determinants are expected to have a detrimental effect on the outcomes. In addition, we wanted to identify the frequency of exposure to (and the nature of) work related traumatic events as well of the occurrence of occupational hazards (physical hazards, violence and situations that increase the risk of burnout) in a sample of Emergency Physicians. We also wanted to explore in this sample what proportion of Emergency Physicians report symptoms of post-traumatic stress, anxiety, depression, somatic complaints, and fatigue at a sub-clinical or clinical level.

## Methods

A total of 346 Emergency Physicians, who attended two national emergency medicine conferences, were approached to take part in the study. Every potential respondent received an invitational letter and an informed consent form during these conferences. The invitational letter stated the study objectives and relevance of the study. After completion of the informed consent form, each respondent was given the choice to receive further correspondence via e-mail or mail. Afterwards, all attending emergency physicians received the questionnaire. Respondents were asked to fill in the questionnaire individually in their leisure time. The questionnaire asked the respondents about work related traumatic events that took place up to 6 months back in time and every respondent was asked to subjectively reflect on how many times these incidents occurred. A reminder was sent 1 month after the start of data collection. Questionnaires could be returned anonymously in a sealed envelope or were completed online protected by a personal code. A total of 181 questionnaires were returned (response rate 52.3 % *n* = 181/346), of which 152 were fully completed (response rate 43.9 %; *n* = 152/346).

### Predictors

#### Socio-demography and personal characteristics

Data was gathered on the socio-demographic status of each respondent, including age, gender, work regime (part- or fulltime), marital status, children living at home, education, seniority, shift work and task diversity (Emergency Station emergency physician, Mobile Urgency Group emergency physician or both).

#### Frequency of exposure to traumatic work related events and life events

Every respondent was asked how many times he/she was confronted with a work-related traumatic event in the past 6 months, and which work-related events had the highest impact. Traumatic work-related events were defined as “a self-experienced traumatizing event, directly related to the work of the EP”. In addition, respondents were asked to indicate whether they had experienced important personal life events during the last year. Conflicts with colleagues or supervisors were excluded from the assessment of work related events, but social support by supervisors and colleagues was measured separately (see below). In addition, the occurrence of situations at work, that can increase the risk of burnout was measured, including work conflict (see below).

#### Occurrence of occupational hazards

In the present study, the occurrence of physical and psychosocial hazards (violence and situations that increase the risk of burnout was measured by means of a yes or no answer to a set of questions that was derived from a list of physical, biological and chemical and psychosocial hazards at the workplace published by Dorevitch S. et al. [6]. The occurrence was explored for the following categories: Infectious disease (blood borne pathogens; hepatitis B; hepatitis C; hepatitis non A - non B; Human Immunodeficiency Virus (HIV); Mycobacterium tuberculosis); Physical hazards (latex allergy; radiation exposure; nitrous oxide); Violence during your work and finally: Situations at work that increase the risk of burnout. (”Have you ever, in your function as emergency physician, experienced one of the following infections/work related problems?”).

#### Social support

Social support by the supervisor and colleagues was measured by means of two subscales of the validated Leiden Quality of Work Questionnaire for Medical Doctors (LQWQ-MD) [19] with a Cronbach’s alpha (α) for each subscale which is used as an estimate of the reliability of a psychometric test [20]. Social support supervisor (α = .91; 4 items) measures perceived social support by the supervisor (e.g. “I feel appreciated by my direct supervisor.”). Social support colleagues (α = .87; 4 items) measures perceived instrumental and emotional support by colleagues (e.g. “My colleagues give me emotional support when I’m having difficulties.”). The items of the LQWQ-MD are occupation specific. In homogeneous samples, occupation specific instruments are preferred over general measures, as they explain more variance in relevant outcome variables. All items are formulated as statements that have to be rated on a 4-point Likert scale, ranging from ‘totally disagree’ to ‘totally agree’.

### Outcome variables

#### Post-traumatic stress symptoms (PTSD)

The validated Impact of Event scale (IES) [21] was used to determine the frequency of post-traumatic stress symptoms and a measure for PTSD, in relation to a recently experienced (in the last 6 months) work-related traumatic event. The Cronbach’s alpha for each subscale and total was also included [20]. The respondent was asked to give a brief description of this event. The IES consists of two subscales: ‘Intrusion’ (α = .90; 7 items), measuring the preoccupation with the traumatic experience, repeated thoughts or nightmares about the experience and a recurrent need to talk about it (e.g. “I had waves of strong feelings about it.”) and ‘Avoidance’ (α = .88; 8 items) measuring self-reported avoidance of certain ideas, feelings, or situations, related to the traumatic event (e.g. “I stayed away from reminders of it.”). All items are rated on a 4-point Likert scale. In the present study, only the total score (sum score of the two dimensions) of the IES was used (α = .94; 15 items), with higher scores being indicative of stronger post-traumatic stress reactions. Normative values for respondents without trauma history, as defined by Briere, were used to compare with the scores of the Emergency Physicians. A cut-off of 20 on the IES was used to differentiate between a mild and a moderate (sub-clinical) level, and a cut-off of 26 was used to distinguish between Emergency Physicians for whom confrontation with traumatic events had a moderate or a major (clinical) impact in terms of symptoms, as a respondent with a score of 26 or higher on the IES has a probability of 75 % or more having post-traumatic stress disorder (PTSD) [22].

#### Psychological distress

Psychological distress was assessed by means of the validated Dutch version of the Brief Symptom Inventory (BSI) with good Cronbach’s alpha (α) for each subscale and sum [20]. Only the subscales ‘anxiety’ (α = .87; 6 items), ‘depression’ (α = .87; 6 items) were used for this study. The BSI has been found to be a good and shorter alternative for the SCL-90R [23, 24]. This instrument assesses the presence of specific symptoms in the past week. Items are rated on a 5-point Likert scale ranging from ‘not at all’ to ‘very much’ for the BSI. Higher scores are indicative of more problems in a specific dimension. ‘Psychological distress’ (α = .93; 12 items) was constructed as a sum score of the dimensions anxiety and depression. Normative values for healthy subjects, as defined by, were used to interpret the score of the Emergency Physicians. The cut-offs defined by De Beurs and Zitman [24] were used to examine how many Emergency Physicians reached a sub-clinical and clinical level of anxiety and depression.

#### Somatic complaints

Somatic complaints were assessed by means of the validated PHQ 15 questionnaire. The total ‘somatization’ score (α = .86; 15 items) was used for this study. This instrument assesses the presence of specific symptoms in the last 4 weeks. All items of the PHQ 15 are rated on a 3-point Likert scale. Higher scores are indicative of more problems in a specific dimension. The cut-offs defined by Kroenke and al. [25] were used to examine how many Emergency Physicians reached a sub-clinical and clinical level of somatic complaints.

#### Perceived fatigue

Fatigue was measured by means of the validated Dutch version of the Checklist Individual Strength (CIS-20R) [26]. This instrument assesses the presence of fatigue symptoms in the past 2 weeks. A Cronbach’s alpha was calculated for the main dimension used in this study [20]. The main dimension of this scale is subjective experience of fatigue (perceived fatigue) (α = .93; 8 items), (e.g. “I’m feeling weak”). For the purpose of this study only this main dimension was used. Items are rated on a 7-point Likert scale ranging from ‘Yes, that's correct’ to ‘No, that's not correct’. A higher score is indicative of a higher level of fatigue. Normative values for healthy subjects were used to interpret the scores of the Emergency Physicians. A cut-off of 35 for the main dimension was used to define clinical levels of fatigue.

#### Job satisfaction

Job satisfaction was measured by means of the job satisfaction subscale (α = .88; 3 items; e.g. “I am satisfied with my job) of the validated Leiden Quality of Work Questionnaire for Medical Doctors (LQWQ-MD) [19]. All items of the questionnaire are formulated as statements that have to be rated on a 4-point Likert scale, ranging from ‘totally disagree’ to ‘totally agree’.

### Statistical methods

The statistical software package for Windows, SPSS 20.0, was used for data analysis. Descriptive statistics (means, standard deviations, skewness and kurtosis) were computed. Pearson correlations, One Way ANOVA and Independent Sample-t tests were calculated between predictors and outcomes. The total score of the impact of event scale (IES), psychological distress (BSI), somatic complaints (PHQ 15), perceived fatigue (CIS 20 R) and job satisfaction (LQWQ-MD) were used as outcomes. Hierarchical regression analyses were conducted to estimate the strength of the association between demographic characteristics (block-1), occurrence of traumatic work events and personal life events (block-2), occurrence of physical hazards/violence/situations that can cause burn-out (block-3) and social support by supervisor/colleagues (block-4) on the one hand and the outcome variables IES total score (PTSD), psychological distress (anxiety and depression), fatigue, somatic complaints and job satisfaction on the other hand. A *p*-value of 0.05 or lower was considered statistically significant.

## Results

### Personal characteristics

The majority of the Emergency Physicians were male (62.3 %; *n* = 95/152). The mean age of the respondents was 44.39 years (SD 9.22). Of these 86 % (*n* = 131/152) had a partner and 69 % (*n* = 105/152) had children living at home. Most of the Emergency Physicians had an emergency specialization degree (78 %; *n* = 118/152). The mean job experience (seniority) in emergency care was 15.44 years (SD 9.40). Almost two thirds of the Emergency Physicians (76.8 %; *n* = 117/152) worked full time (16 shifts of 12 h/day) and 84.1 % (*n* = 128/152) worked in changing shifts, including night shifts. Two thirds worked in a non – university hospital (73.5 %; *n* = 112/152). All of the respondents participated in an in-hospital emergency care, but a major proportion also participated as Emergency Physicians in emergency out-hospital services as a MUG-physician (Mobile Urgency Group). Furthermore, 87.4 % (*n* = 133/152) were members of an in-hospital resuscitation team.

### Frequency of exposure to and type of traumatic events

75 % (*n* = 114/152) of the respondents reported one or more traumatic events in the last 6 months. Only one quarter of the respondents reported no confrontation with a traumatic event in the last 6 months. Of those reporting a traumatic event 10 % (*n* = 15/152) reported only one traumatic event, 23 % (*n* = 35/152) reported two or three of these events, 17 % (*n* = 26/152) reported four or five events and 25 % (*n* = 38/152) reported more than five traumatic events.

Table 1 shows the top 10 nature of the traumatic events the respondents were confronted with in the previous 6 months, and the percentage of respondents who mentioned an event as the most distressing one. ‘Dealing with the sudden unexpected death of a young person’ and ‘traumatic accident/disease involving a young person’ was considered to be the most distressing events by 26 % and 10 % of the Emergency Physicians, respectively.

**Table 1:** Nature of traumatic events in order of the percentage of respondents (*N* = 152) mentioning an event as the most distressing one

Top 10 of traumatic events reported by Emergency Physicians		*N* (%)
1.	Dealing with sudden death of a young person	40 (26)
2.	Traumatic accident/disease involving a young person	15 (10)
3.	Dealing with severe injuries	14 (9)
4.	Dealing with death after resuscitation or resuscitation of a young person	14 (9)
5.	Missed diagnosis	12 (8)
6.	Dealing with suicide	9 (6)
7.	Dealing with death in general	9 (6)
8.	Inability to help chronically ill patients	6 (4)
9.	Dealing with aggression, violence and threat	5 (3)
10.	Lawsuits	5 (3)

The number and percentage of respondents reaching sub-clinical and clinical cut-offs for the different outcomes can be found in Table 2.

**Table 2:** Comparison of the outcome variables for the respondents of this study (*N* = 152) with normative data and the number (percentage) of respondents reaching the sub-clinical and clinical cutoffs for PTSD, anxiety, depression, somatic complaints and the clinical cutoff for perceived fatigue

Outcome variable	Means (SD)	Sign.	Cutoff	Subclinical level	Cutoff	Clinical level
PTSR (IES)				*N* (%)		*N* (%)
Emergency Physicians Missing 2 resp.	10.98 (13.97)	**	20–25	30 (19.8 %)	≥26	22 (14.5 %)
Normative sample	8.10 (12.30)					
Anxiety (BSI)				*N* (%)		*N* (%)
Emergency Physicians	0.61 (0.72)	*	0.42–1.37	68 (44.7 %)	≥1.38	16 (10.5 %)
Normative sample	0.33 (0.51)					
Depression (BSI)				N (%)		N (%)
Emergency Physicians	0.55 (0.71)	*	0.36–1.73	64 (44.1.x %)	≥1.74	12 (7.9 %)
Normative sample	0.31 (0.53)					
Somatic complaints (PHQ 15)				*N* (%)		*N* (%)
Emergency Physicians	5.99 (4.69)	*	5–9	82 (53.9 %)	≥10	26 (17.1 %)
Normative sample	3.80 (4.10)					
Perceived fatigue (CIS-20R)						*N* (%)
Emergency Physicians	29.63 (16.73)	*			>35	52 (34.2 %)
Normative sample	17.30 (10.10)					

Abbreviations: ** Correlation is significant at the 0.01 level.* at the 0.05 level, resp. = respondents

Bold values denote significant treatment differences

### Post-traumatic stress symptoms

The mean score for the Emergency Physicians on the IES was significantly higher than the normative sample. In accordance with Corneil et al. [27] a total score of >20 was used as a sub-clinical cut-off and a score of 26 or higher was used as a clinical cut-off, considered to be indicative of traumatic stress symptoms with likelihood of Post-Traumatic Stress Disorder (PTSD). In the present study, 19.8 % of the respondents scored above the sub-clinical cut-off, and 14.5 % reached clinical levels indicative of PTSD.

### Anxiety and depression

According to the available cut-offs, defined by De Beurs and Zitman [24], 34.2 % of our population reached a subclinical and 10.5 % a clinical level of anxiety. For depression 34.2 % met the subclinical level and 7.9 % the clinical level.

### Somatic complaints

For ‘somatic complaints’, measured by the PHQ15, 36.8 % of the respondents exceeded sub-clinical levels while 17.1 % scored above the clinical cut-off point [28].

### Perceived fatigue

The mean score on the main dimension of the CIS-20R (perceived fatigue scale) was significantly higher than the normative sample of healthy subjects [26]. In the present study, 34.2 % of the respondents reached the clinical cut-off score.

### Correlations

The correlations between independent and dependent variables are reported in Table 3, together with descriptive data for each variable and a Cronbach’s alpha (α) for each scale [20]. Correlations between the independent variables were all lower than .70, except for the correlation between age and job seniority (*r* = .96). Job seniority was therefore excluded from the hierarchical regression analyses.

**Table 3:** Inter-correlations (Pearson correlation coefficients) for age, seniority, the occurrence of physical hazards, violence, situations that can cause burnout, social support supervisor and social support colleagues and outcome variables

		α	1	2	3	4	5	6	7	8	9	10	11	12
1.	Age	-	-											
2.	Seniority	-	**,96** ^**^	-										
3.	Occurrence Physical Hazards	-	-,11	-,11	-									
4.	Occurrence Violence	-	**,16** ^*^	**,16** ^*^	**,28** ^**^	-								
5.	Occurrence Burnout	-	,10	,11	**,19** ^*^	**,25** ^**^	-							
6.	Supervisor support	.91	-,09	-,09	-,01	-,09	**-,31** ^**^	-						
7.	Colleagues support	.87	-,02	-,01	,08	**-,16** ^*^	**-,33** ^**^	**,62** ^**^	-					
8.	IES Total (PTSR)	.94	,06	,02	,07	**,27** ^**^	**,42** ^**^	**-,23** ^**^	**-,31** ^**^	-				
9.	Psychological distress (BSI)	.93	**-,18** ^*^	**-,19** ^*^	,14	**,25** ^**^	**,47** ^**^	**-,21** ^**^	**-,31** ^**^	**,59** ^**^	-			
10.	Somatization (PHQ 15)	.86	-,11	-,12	,09	**,24** ^**^	**,47** ^**^	-,13	**-,38** ^**^	**,53** ^**^	**,69** ^**^	-		
11.	Subjective Fatigue (CIS-20R)	.93	-,09	-,11	**,24** ^**^	**,43** ^**^	**,46** ^**^	**-,16** ^*^	**-,32** ^**^	**,38** ^**^	**,64** ^**^	**,69** ^**^	-	
12.	Job satisfaction (LQWQ-MD)	.88	,00	-,03	-,01	**-,20** ^*^	**-,50** ^**^	**,46** ^**^	**,56** ^**^	**-,26** ^**^	**-,38** ^**^	**-,39** ^**^	**-,53** ^**^	1
		M	44,3	15,3	0,3	0,5	0.2	11.0	11.7	22.9	19.0	20.9	4.5	5.88
		D	9,2	9,4	0,5	0,5	0.4	3.2	2.5	9.2	8,2	5.0	1.95	2.2

*Abbreviations*: *α* Cronbach’s Alpha, Correlation significant *p ≤ .05. ** *p* ≤ .01 level (2-tailed), *LQWQ-MD* Leiden Quality of Work Questionnaire for Emergency Physicians, *IES* impact of event scale, *CIS-20R* Checklist Individual Strength, *PHQ 15* Somatization PHQ15 Checklist, *PTSR* Posttraumatic stress reaction, *BSI* Brief Symptom Inventory, the data in bold have significant correlation

### Regression analyses

Hierarchical regression analysis was performed to estimate the strength of the association between demographic characteristics (block-1), frequency of exposure (block-2), exposure to traumatic events (block-3) and social support (block-4) on the one hand and each of the outcome variables: IES total, psychological distress, perceived fatigue, somatic complaints and job satisfaction on the other hand. The results of these hierarchical regression analyses are reported in Table 4.

**Table 4:** Summary of hierarchical regression analysis: personal characteristics (block 1), exposure to traumatic events (block 2), occurrence (block 3) and social support (block 4) as predictors of post-traumatic stress distress, psychological distress, perceived fatigue, somatic complaints, job satisfaction

	IES total			Psychological distress			Perceived fatigue			Somatic complaints			Job Satisfaction		
	Δ*R* ^2^	*β*	Sign	Δ*R* ^2^	*β*	Sign	Δ*R* ^2^	*β*	Sign	Δ*R* ^2^	*β*	Sign	ΔR2	β	Sign
Block 1: demographics	0.00			0.04			0.03			0.03			0.01		
Age		,05	n.s		**-,17**	*		-,11	n.s		**-,17**	*		-,02	n.s
Workschedule		,02	n.s		,08	n.s		,13	n.s		,05	n.s		-,11	n.s
Block 2: Traumatic events	0.12			0.06			0.02			0.02			0.01		
Age		,09	n.s		-,14	n.s		-,10	n.s		-,15	0.07		-,03	n.s
Workschedule		,01	n.s		,07	n.s		,12	n.s		,04	n.s		-,10	n.s
Traumatic events (work)		**,33**	***		**,25**	***		,14	n.s		,15	0.06		,01	n.s
Life events (personal life)		-,12	n.s		-,05	n.s		,06	n.s		-,02	n.s		,11	n.s
Block 3: occurrence	0.15			0.23			0.28			0.23			0.28		
Age		,02	n.s		**-,22**	***		**-,19**	**		**-,24**	***		,07	n.s
Workschedule		,03	n.s		,01	n.s		**,15**	*		,07	n.s		**-,14**	*
Traumatic events (work)		**,23**	***		,14	0.06		,01	n.s		,04	n.s		,09	n.s
Life events (personal life)		-,12	n.s		-,05	n.s		,05	n.s		-,02	n.s		,11	n.s
Occurrence physical total		-,07	n.s		-,03	n.s		,05	n.s		-,10	n.s		**,15**	*
Occurrence violence		**,17**	*		**,17**	*		**,29**	***		**,18**	*		**-,17**	*
Occurrence situations that can cause burnout		**,35**	***		**,44**	***		**,38**	***		**,44**	***		**-,50**	***
Block 4: social support	0.02			0.02			0.03			0.07			0.16		
Age		,03	n.s		**-,21**	***		**-,17**	*		**-,21**	***		,06	n.s
Workschedule		,02	n.s		,09	n.s		**,14**	*		,06	n.s		-,11	0.09
Traumatic events (work)		**,23**	***		**,14**	*		,01	n.s		,05	n.s		,06	n.s
Life events (personal life)		-,12	n.s		-,05	n.s		,04	n.s		-,03	n.s		,11	n.s
Occurrence physical total		-,04	n.s		-,00	n.s		,10	n.s		-,03	n.s		,08	n.s
Occurrence violence		,15	0.07		**,15**	*		**,27**	***		**,15**	*		-,11	0.09
Occurrence situations that can cause burnout		**,30**	***		**,39**	***		**,34**	***		**,38**	***		**-,34**	***
Social support supervisor		,01	n.s		,03	n.s		,13	n.s		**,22**	**		,14	n.s
Social support colleagues		-,17	0.07		-,17	0.07		**-,23**	**		**-,36**	***		**,33**	***
	*R* ^2^ model	0.29		*R* ^2^ model	0.35		*R* ^2^ model	0.36		*R* ^2^ model	0.35		*R2* model	0.46	
	Adj *R* ^2^ model	0.25		Adj *R* ^2^ model	0.31		Adj *R* ^2^ model	0.33		Adj *R* ^2^ model	0.31		Adj *R2* model	0.43	

*Abbreviations*: *ΔR* ^*2*^ change in R^2^ values from one model to another, *R* ^*2*^ *model* R^2^ values in one model, *Adj R* ^*2*^ *model* adjusted R^2^ values in one model, *β* beta resulting standardized regression coefficients, *Sign* significant, *n.s* not significant, * Correlation is significant at the 0.05 level, ** Correlation is significant at the 0.01 level,*** Correlation is significant at the 0.001 level, IES total = post-traumatic stress distress, the bold data are significant

With regard to the IES total score, measuring the severity of post-traumatic stress reactions, the regression model including only personal characteristics (block-1) did not significantly differ from the null model. Frequency of exposure to traumatic events (block-2) explained an important part of the variance (15 %). Social support (block-4) explained an extra 2 % of the variance. Better perceived social support from colleagues was associated with less PTSS. The final model, including all four blocks, explained 29 % of variance in PTSS.

With regard to psychological distress, age explained a small part of the variance (4 %) as well as frequency of traumatic events (6 %), while most of the variance was explained by occurrence of violence and especially situations that increase the risk of burnout. The final model explains 35 % of the variance in psychological distress.

With regard to fatigue personal characteristics explained a small part of the variance, but frequency of traumatic events (block-2) did not significantly contribute. Occurrence of occupational hazards (block-3) explained the major part of the variance (28 %). Finally, social support by colleagues added an extra 3 % to the explained variance. Adequate social support by colleagues was associated with less perceived fatigue. The final model, including all four blocks, explained 36 % of the variance.

With regard to perceived somatic complaints, age explained a small part (3 %) of the variance, but frequency of traumatic events (block-2) did not significantly contribute (3 %). Occurrence of occupational hazards (block-3) explained the largest part of the variance (23 %). Social support (block-4) added an extra 7 % of explained variance. Adequate social support by colleagues was associated with less complaints, in contrast to social support from the supervisor. The final model, including all four blocks, explained 35 % of the variance in somatic complaints.

With regard to job satisfaction, age explained a very small part of the variance (1 %), but frequency of traumatic events (block-2) did not significantly differ from the null model. Occurrence of occupational hazards (block-3) explained the largest part of the variance (28 %). Social support (block-4) added an extra 16 % of explained variance. Adequate social support by colleagues was associated with high job satisfaction, in contrast to social support from the supervisor. The final model, including all four blocks, explained 46 % of the variance in job satisfaction.

## Discussion

### The most interesting study results

Of the EPs who participated in the present study 75 % reported confrontation with one or more traumatic events over the last 6 months. A British study in ambulance workers, consisting of EPs and ambulance personnel found that 82 % of the respondents had experienced a disturbing event in the previous 6 months [14]. In conclusion EPs are, according to our and other studies, confronted on a regular basis with significant, potentially traumatizing work related events [9, 29, 30]. There is a higher perception of traumatic events in older EPs.

We ascertained that 36 % (*n* = 55/152) of the EPs find dealing with sudden death of a young person and traumatic accident/disease involving a young person the most traumatic experience during their work activity. Three quarter of these EPs have children of their own (*n* = 29/55). It is also clear that the female EPs, with an average age of 41 years old and whom have children of their own (*n* = 29/55), rate this experience as most traumatic in their work environment. These findings are supported by the results of other studies [31–33].

The results also show that a substantial part of the EPs exceed sub-clinical levels of post-traumatic stress (19.8 %), anxiety (44.7 %), depression (42.1 %), and somatic complaints (53, 9 %). Moreover, about one third of the respondents reach clinical levels of fatigue, while one out of seven EPs reach a clinical level of posttraumatic symptoms, called Post Traumatic Stress Disorder (PTSD) and 17,1 % meet a clinical level of somatic complaints. These findings are comparable to those of other studies [9, 34].

The main aim of the study was to determine the impact of demographic characteristics, frequency of confrontation with work related traumatic events and life events, the occurrence of occupational physical and psychosocial hazards and perceived social support to PTSS, psychological distress (anxiety and depression), somatic complaints, fatigue and job satisfaction in Emergency Physicians. The results of the present study show that frequency of exposure to traumatic (work) events contributes next to occurrence of situations that increase the risk of burnout to the explanation of variance in posttraumatic stress and psychological distress. Moreover the occurrence of occupational hazards is strongly related to all outcomes. Especially occurrence of situations that increase the risk of burnout seems to have a major impact on all outcomes including job satisfaction, while occurrence of violence contributes especially to psychological distress and perceived fatigue. As such psychosocial occupational hazards seem to have much more impact than physical hazards. Social support by colleagues seems to buffer to some extent fatigue and somatic complaints and especially appears to reduce the potential detrimental effects of confrontation with situations that increase the risk of burnout on job satisfaction. Lack of social support is a known predictor of occupational stress in emergency care workers [35, 36]. In contrast however, good social support of colleagues at work, as we found in our study, can facilitate the recovery process after confrontation with traumatic events and occupational hazards [37, 38].

### Implications for practice

The novelty of this study is that it explores the effect of specific determinants of PTSS, psychological distress, fatigue, somatic complaints and job satisfaction in Emergency Physicians. Especially confrontation with patient violence and situations that can increase the risk of burnout seem to be detrimental. In contrast, social support by colleagues seems to have a beneficial effect on several outcomes. Post death debriefing and seeking comfort with colleagues, can help to overcome such an experience. Rest, distraction and relaxation can also help increase the positive attitude to the EPs work and recognize that death is part of the job in the Emergency Medicine Practice [39]. The study also points at alarmingly high (sub) clinical stress consequences of confrontation with traumatic work related events and psychosocial occupational hazards in Emergency Physicians, which may also lead to adverse effects on the quality of care [40]. Regular screening as well as mentoring of high-risk emergency physicians should therefore be considered, particularly following a major traumatic event or a cumulative occurrence of traumatic events [41]. In addition, as confrontation with violence seems to explain parts of the variance in several outcomes, a training of EPs in dealing with violence is indicated. There is evidence that training (learning to anticipate, recognize and respond to violence) and techniques of dealing with aggressive patients, including eliminating solo interventions, may prevent injury in health care workers [42]. Furthermore, as confrontation with situations that increase the risk of burnout seems to be the strongest predictor of all outcomes, burnout prevention and treatment should be a priority in emergency medicine. There is a lot of evidence that burnout in emergency health care workers is strongly related to job demands, lack of control and social support and to organizational stressors. Screening emergency units systematically on these predictors in view of interventions, e.g. by means of the LQWQ/MD, is thus an important priority [43]. As especially social support from colleagues seems to buffer detrimental effects in several outcomes, efforts to create a supportive, communicative work environment are very important. Still apart from this, the medical staff should thus be the first in line to support a colleague who was confronted with important traumatic work related events or other important stressors at work. Training the staff in supportive skills and provision of back-up support by an experienced clinical psychologist are thus also important in the prevention of detrimental consequences as previously mentioned in both American and European studies [15, 16].

### Strengths and limitations of the study

The response rate and the relatively large, representative sample of Emergency Physicians are important strengths of this study in comparison to other studies. This study also provides data on the frequency and the nature of exposure to traumatic events as well as the percentages of the EPs who meet (sub) clinical levels for PTSS, anxiety, depression, somatic complaints fatigue and job satisfaction.

The study has however also several limitations. As far as the main research question is concerned, due to the cross-sectional design of the study, it is difficult to determine cause effect relationship. In addition, frequency of exposure to traumatic work related events and occurrence of occupational hazards was not measured in real time, but retrospectively. Another shortcoming is that we excluded conflict with supervisors and colleagues from the assessment of work-related traumatic events. Next, although most of the measures that we used were validated, we also used a self-developed questionnaire that was based on a previous study to measure occupational hazards [44]. Finally, the relatively high prevalence of (sub) clinical levels in various outcomes can also be explained by other predictors, such as personal problems and work related conditions, than the ones included in this study.

Despite certain limitations, this study is original since it identifies important specific predictors of post-traumatic stress, psychological distress, fatigue and other somatic complaints as well as job satisfaction in emergency physicians. All of these predictors need attention and a more of the, can be influenced by structural and managerial initiatives.

Further research should carry out assessment of traumatic events and occupational hazards in real time, use a repeated measures design to assess the stability of the relationship between predictors and outcomes over time and include additional predictors such as job demands, job control, and organizational stressors such as quantity and quality of staffing, availability of necessary equipment, detrimental communication, work conflicts and social harassment at work.

## Conclusion

Emergency physicians are routinely confronted with work related traumatic events and hectic work conditions. The results of this study show that levels of anxiety, depression and somatic complaints and post-traumatic stress reactions are indeed high in Emergency Physicians. Occurrence of violence is related to psychological distress, perceived fatigue and somatic complaints, while occurrence of situations that increase the risk of burnout is related to all outcomes. Finally, the positive effect of social support of colleagues has a buffering effect on most outcomes.

Screening EPs regularly on important outcomes including burnout in order to provide timely professional and social support for EPs at risk; offering training in dealing with violence, stress management and stress reduction skills; providing debriefing by colleagues and if necessary by a clinical psychologist after confrontation with an important traumatic work event, providing time for rest and relaxation when necessary and creating a supportive work environment, by increasing supportive skills and creating a good team spirit in the staff, are important interventions that can prevent or reduce detrimental consequences in EPs.

## Ethical approval

Approval from the Ethical Committee of AZ St. Dimpna Geel (Erica) for this study was obtained. Confidentiality was guaranteed to all participants. Informed consent was signed by each respondent before data collection.
